# Translation into Chinese of: "Changes to publication requirements made at the XVIII International Botanical Congress in Melbourne - what does e-publication mean for you?". Translated by Li-Bing Zhang 在墨尔本召开的第18届国际植物学大会做出的有关名称发表要求的变化 —电子出版物对你意味着什么？

**DOI:** 10.3897/phytokeys.6.1984

**Published:** 2011-09-14

**Authors:** Sandra Knapp, John McNeill, Nicholas J. Turland

**Affiliations:** 1 Department of Botany, The Natural History Museum, Cromwell Road, London SW7 5BD, UK The Natural History Museum London United Kingdom; 2 Royal Botanic Garden, Edinburgh, 20A Inverleith Row, Edinburgh EH3 5LR, UK Royal Botanic Garden Edinburgh United Kingdom; 3 Missouri Botanical Garden, PO Box 299, St Louis, MO 63166-0299, USA Missouri Botanical Garden St. Louis United States of America

## Abstract

《国际植物命名法规》的修订由每六年一次的国际植物学大会(IBC)命名分会来决定。第18届国际植物学大会在澳大利亚墨尔本举行；命名分会于2011年7月18日至22日召开，其决议获得7月30日的全体会议通过。“墨尔本法规”有几个重要的变化，将影响新名称的发表。这些变化中的两个将在“墨尔本法规”出版前的几个月，即于2012年1月1日起生效。通过以移动文档格式(Portable Document Format; pdf)在线发表的具有国际标准连续出版物号(ISSN)或国际标准图书编号(ISBN)的电出版物，将构成有效发表。新分类群名称的合格发表所必须的拉丁文描述或特征集要将更改为拉丁文或英文描述或特征集要。此外，自2013年1月1日起，被处理为真菌的生物的新名称必须在原始资料(某一名称合格发表时与之有关的所有资料)中引证一个由一家公认的存储库(例如MycoBank)签发的标识符，才构成合格发表。本文提供了有关电子出版物的新规则的草案文本，并概述了相应的最佳做法。

为便于新的《国际藻类、真菌和植物命名法规》中的变化得到广泛的了解，本文将发表在*BMC Evolutionary Biology, Botanical Journal of the Linnean Society, Brittonia, Cladistics, MycoKeys, Mycotaxon, New Phytologist, North American Fungi, Novon, Opuscula Philolichenum, PhytoKeys, Phytoneuron, Phytotaxa,*《植物分类与资源学报》*, Systematic Botany*和*Taxon*。

## 序言

于2011年7月在澳大利亚墨尔本召开的第18届国际植物学大会通过的《国际植物命名法规》(现称为《国际藻类、真菌和植物命名法》规)有两个重要的变化，并将于2012年1月1日生效。这些变化将影响到每一个发表相关名称的人。由于“墨尔本法规”大约要等到2012年中才能出版，我们认为将这些变化，特别是那些有关在电子媒体中有效发表的变化(规则29，30，31)，做一概述，将对我们的读者有所裨益。有关在墨尔本接受的所有变化的一个简明报告，可参阅 McNeill et al. (2011)。

本文提供了修订后的有关有效发表的规则、注释和辅则的草案，以帮助编辑和出版商建立为实施本法规的最佳做法。我们这里还概述了这些变化并不包含的意思，以指导那些希望通过电子方式发表新名称和指定模式的人们。我们促请读者阅读电子发表特别委员会的报告 Chapman et al. 2010 以及国际植物学大会之前的相关提案。有关法规修订的理由在那里有详细的陈述。

## 修订后的规则29, 30和31, 和辅则29A, 30A及31A的草案

在这里，我们复制了所有相关的规则、注释和辅则(省去例子)，黑体字指法规中新的变化。这里的措辞只是临时性的，印刷版的“墨尔本法规”的内容将由2011年12月召开的法规编辑委员会会议来决定。

### 规则29

*29.1.* 根据本法规，只有将印刷品向一般公众发行(通过出售、交换或赠送)，或至少分送给具有普通植物学家可使用的图书馆的植物学研究机构，这样的发表才是有效发表(effective publication)。**通过以移动文档格式(Portable Document Format)(pdf；也见规则29.3及辅则29A.1)在线发表的具有国际标准连续出版物号(International Standard Serial Number; ISSN)或国际标准图书编号(International Standard Book Number; ISBN)的电子发行，也是有效发表。**在公共会议上对新名称的交流，或将名称置于对公众开放的标本馆或植物园，或发行由文稿(manuscript)、打字稿(typescripts)或其他未发表的材料(unpublished material)制成的微缩胶片(microfilm)，或**通过上述电子发表方式之外的**电子发表，均为无效发表。

***29.2.* 就本规则的实际应用而言，“在线”指的是可通过万维网(World Wide Web)**访问的。

***29.3.* 如果今后移动文档格式(pdf)被取代，可以接受由总委员会(见第三部分)通报的下一个国际标准格式。**

***29.4.* 一个电子出版物首次发行以后，其内容便不能更改。任何更改都是无效发表。对其更正或修改必须独立地发表才构成有效发表。**

### 辅则29A

[现有辅则被下面辅则取代]

***29A.1.*以移动文档格式(pdf)的电子发表应遵照pdf/A存档标准(国际标准化组织(ISO)19005)。**

***29A.2.*在切实可行的情况下，作者在发表存档的出版物时，应该最好满足以下条件(也见辅则29A.1):**

**(*a*)材料应存放在多个值得信赖的在线数字资源库，如国际标准化组织(ISO)认证的存储库;**

**(*b*)数字资源库应放置在世界上多个地区，最好在不同的大洲;**

**(*c*)印本应存放在世界上多个地区、最好在不同的大洲的图书馆。**

### 规则30

***30.1.* 2012年1月1日前电子材料的分发并不构成有效的发表。**

***30.2.* 如果存在跟电子出版物相关的证据或有电子出版物内部的证据显示，该电子出版物仅仅是一个初级的版本或将要被一个出版者所认为的最终版本所取代，该电子出版物便不构成有效发表。在这种情况下，只有最终版本才是有效发表。**

*30.3.* 1953年1月1日之前的擦不掉的手写体(indelible autograph)出版物是有效发表。其后出现的擦不掉的手写体为无效发表。

*30.4.* 就本规则的具体应用而言，擦不掉的手写体是指通过机械或图像过程(例如平版印刷，胶印，或金属蚀刻)复制的手写材料。

*30.5.* 1953年1月1日或之后发行的商业目录(trade catalogue)或非学术性报纸，以及1973年1月1日或之后发行的种子交换目录(seed-exchange list)，均不构成有效发表。

*30.6.* 在1953年1月1日或之后随腊叶标本(exsiccatae)而被分发的印刷品不构成有效发表。

注释1. 如果该印刷品在随腊叶标本之外也有分发，则构成有效发表。

*30.7.* 在1953年1月1日或之后，指明为提交给大学或其它教育机构而为获得某种学位的、独立的、非系列的毕业论文的发表，是无效发表，除非论文中清楚地说明(指本法规中有效发表的必要条件)或其它的内部证据(internal evidence)显示其作者或出版者视它为有效发表。

注释2. 国际标准图书编号(International Standard Book Number; ISBN)的存在，或在原始印刷版中指出了有关印刷厂、出版者或发行者的名字，均被视为相关著作有意为有效发表的内部证据。

### 辅则30A

***30A.1.*** 同一电子出版物的初级和最终版本在第一次发行时，应清楚地表明它们的版本。

*30A.2.* 本法规强烈地建议，作者应竭力避免在任何临时性的印刷品(ephemeral printed matter)中、特别是那些印数有限和不定的印刷品中发表新名称和新分类群的描述或特征集要(命名新材料nomenclatural novelties)。这样的印刷品内容的持久性可能会受到限制，其有效发表的拷贝数亦不明显，或者一般公众不太可能接触到。作者也应避免在通俗刊物(popular periodicals)、文摘杂志(abstracting journals)或勘误表(correction slip)上发表新名称和描述或特征集要。

*30A.3.* 为提高时间和地点方面的可用性，发表命名新材料的作者应尽量选择经常发表分类学文章的期刊。**否则，应将其文章的拷贝**(**无论是印本发表或电子发表**)**寄给适当的与分类群相关的索引中心(indexing centre)。只有印本发表的文章应**存于世界上至少10个或最好更多的植物学或其它一般的公共图书馆。

*30A.4.* 作者和编辑最好在摘要(abstract)或概要(summary)中提及所发表的命名新材料，或将其列在出版物的索引中。

### 规则31

*31.1.* 有效发表的日期是，根据规则29和30的规定，印刷品**或电子件**可被使用的日期。如果没有证据证明不同，印刷品**或电子件**上的日期必须被接受为正确的日期。

[现有的注释1被以下规则取代]

***31.2.* 当一个出版物同时发行在印刷版和电子版上，必须视印刷版和电子版的有效发表日期相同，除非根据规则31.1. 印刷版和电子版的有效发表日期不同。**

*31.3.* 当期刊或其它出售品的抽印本被提前发行时，抽印本上的日期为有效发表日期，除非有证据显示该日期是错误的。

### 辅则31A

*31A.1.* 出版商或其代理将印刷品交付给平常的投递人向公众发行的日期，应被接受为该印刷品的有效发表日期。

## 最佳做法

新名称的作者、编辑和出版商都将确保含新名称的出版物符合“墨尔本法规”，以便其中的名称是有效发表。我们建议，含网络版的学术期刊或专著系列和书籍的作者与相关编辑沟通，使最佳做法可以在整个领域尽快建立起来。在有一段时间以来，许多出版商都精心讨论过有关新材料的电子发表问题(见 Knapp and Wright 2010，*PLoS One*指南[http://www.plosone.org/static/policies.action#taxon])。对法规的这些修改使其有效地发挥作用，已势在必行。

我们认为，有些做法将有助于符合“墨尔本法规”的电子发表新材料的开始阶段：

• 在每篇文章的醒目位置注明出版日期(象许多杂志所为，例如*New Phytologist*或*Nature*)。
• 如果早期网络版不是最终版(因而不是有效发表的地方)，应在醒目位置注明这一事实(例如*American Journal of Botany*)。
• 突出显示每篇文章所在的出版物的ISSN或ISBN，将有助于索引编写者确定有关名称是否为有效发表。
• 发表在参与CLOCKSS系统(描述见 Knapp and Wright 2010)或其他国际存档和保存系统的期刊(或专著系列)上的文章，将确保其长期归档。
• 通过电子方式发表新名称的作者，应采纳辅则30A.3的建议而提醒适当的索引中心。这将有助于索引编写者意识到其通过电子方式发表的名称。

## 这些变化不意味着什么

尽管新的规则和辅则使用PDF和PDF / A术语，这并不意味着，为了有效发表就只能发行这种格式。例如，一些在线期刊发行超文本标记语言(Hypertext Markup Language; HTML)格式的论文，同时还有一个PDF的版本。在这种情况下，PDF版将是有效发表。如果将来PDF格式被取代，植物命名总委员会将通报一个新的国际标准格式的接受情况。这一规定意味着，新材料的作者和使用法规的人们会及时了解到该领域的进展情况，并保护法规不至于过时。

根据“墨尔本法规”，使用下列电子出版方式不构成新材料的有效发表：

• 网页上的发表或在因特网上出现的短暂文件(发放ISSN号有严格的标准–见[http://www.issn.org])。
• 没有ISSN号或e-ISSN号的期刊上的发表。
• 没有ISSN号或e-ISSN号的书籍中的发表。

获得植物学大会通过的辅则虽建议植物学家们将任何电子出版物的电脑打印件保存在图书馆里，但该辅则并没设定标准做法或需图书馆馆员遵从的规程。图书馆馆员本身处在出版模式之间的复杂的过渡区(Johnson and Luther 2007)。植物学家可能会发现，如果要保存的量很大，图书馆馆员不愿或无法收存个人单篇论文的电脑打印件。

## 法规中另外两个重要的有关名称发表的变化

在墨尔本获得通过的从2012年1月1日起生效的法规的第二个变化是，法规所管理的所有生物的新分类群名称的合格发表所需的描述或特征集要可以是英文或拉丁文。这是目前对发表植物化石名称的规定，但所有非化石分类群的新名称的发表，目前都需要拉丁文描述或特征集要(真菌和植物从1935年1月1日开始，藻类[包括根据该法规处理的蓝藻]从1958年1月1日开始)。这一变化跟学名的形式没有关系。学名将继续为拉丁词或被处理为拉丁词。当然，至于期刊要求描述或特征集要为英文和/或拉丁文，则由这些期刊的编辑决定。

直到2013年1月1日才生效(不是如Miller et al. 2011 所报道的2012年1月1日)的第三个在墨尔本获得通过的有关名称发表的法规的变化是，作为合格发表的附加要求，被处理为真菌的生物的所有新名称必须在原始资料(某一名称合格发表时与之有关的所有资料)中引证一个由一家公认的存储库(例如MycoBank [http://www.mycobank.org/])签发的标识码。这将另行公布。

2013年1月1日或之后的真菌新名称的独特标识码的规定并不适用于植物或藻类；这些类群的新名称的作者不需要从索引中心申请生命科学标识码 (Life Science Identifiers; LSIDs)或其他标识码。

## References

[B1] Chapman AD Turland NJ Watson MF (Eds) (2010) Report of the Special Committee on Electronic Publication. Taxon 59: 1853-1862.

[B2] Johnson RK Luther J (2007) The E-Only Tipping Point for Journals: What’s Ahead in the Print-to-Electronic Transition Zone. Association of Research Librarians, Washington DC.

[B3] Knapp S Wright D (2010) E-publish or perish? In: Polaszek A (Ed). Systema Naturae 250 – the Linnaean Ark. Taylor and Francis, London: 83-93. doi: 10.1201/EBK1420095012-c8

[B4] McNeill J Turland NJ Monro A Lepschi BJ (2011) XVIII International Botanical Congress: preliminary mail vote and report of Congress action on nomenclature proposals. Taxon 60: 1-14.

[B5] Miller JS Funk VA Wagner WL Barrie F Hoch PC Herendeen P (2011) Outcomes of the 2011 Botanical Nomenclature Section at the XVIII International Botanical Congress. PhytoKeys 5: 1-3. doi: 10.3897/phytokeys.5.1850 PMC3174450 22171188

